# Italian radiologists and dual-energy CT: a state of the art from a shared document by the computed tomography subspecialty section of the Italian society of radiology

**DOI:** 10.1007/s11547-025-02044-5

**Published:** 2025-07-15

**Authors:** Maria Antonietta Mazzei, Alfonso Cerase, Giulio Bagnacci, Armando Perrella, Nunzia Di Meglio, Francesco Giuseppe Mazzei, Luca Volterrani, Giulia A. Zamboni, Michaela Cellina, Susanna Guerrini, Giuseppe Minetti, Laura Maria Cacioppa, Giorgio Ascenti, Chiara Floridi, Andrea Giovagnoni, Italian Society of Medical and Interventional Radiology partecipants, Teresa Arcadi, Giovanni Maria Argiolas, Francesco Marcello Arico, Velio Ascenti, Luca Ausili Cefaro, Vittoria Balletta, Claudia Barillà, Stefano Bastaniello, Eugenio Belatti, Raffaele Bisogno, Giovanni Bonenti, Antonio Bottari, Pasquale Bufano, Guido Buonomenna, Cristina Calandra, Tanio Campagnuolo, Delia Campanella, Roberto Cannella, Danilo Caudo, Giuseppe Cicero, Giuseppe Cittadini, Valeria Consoli, Tommaso D’Angelo, Annemilia del Ciello, Vito Di Martino, Margherita Di Stefano, Alessandra Farchione, Antonio Pio Francioso, Barbara Frittoli, Rachele Fruzza, Valeria Garufi, Chiara Gennari, Francesco Gentili, Ivan Gigantelli, Mariangela Iodice, Anna Rita Larici, Enrica Liguori, Stefano Lofino, Antonio Lo Tito, Nicola Maggialetti, Francesca Menchini, Cinzia Micheli, Vittorio Miele, Nicola Migliaccio, Bruno Minopoli, Ilaria Monteleone, Emanuele Neri, Luca Panebianco, Fabio Perotto, Matteo Pignatelli, Andrea Pisano, Gabriele Polonara, Giuseppe Posillico, Sergio Racchiusa, Lorenzo Santini, Francesco Secchi, Cristian Sica, Salvatore Silipigni, Carmelo Sofia, Alberto Stagno, Silvia Storer, Corrado Tagliati, Ilaria Tanga, Francesco Testa, Michele Tonerini, Giorgio Torrigiani, Chiara Torrisi, Francesco Toscano, Silvia Tresoldi, Umberto Tupputi, Francesco Verde, Carlo Zanolini, Fabio Zecca

**Affiliations:** 1 Italian College of Computed Tomography of Italian Society of Medical and Interventional Radiology (SIRM), 20122 Milan, Italy; 2 Unit of Diagnostic Imaging, Department of Medical, Surgical and Neuro Sciences and of Radiological Sciences, University of Siena, Azienda Ospedaliero-Universitaria Senese, Viale Mario Bracci 11, 53100 Siena, Italy; 3 Diagnostic and Therapeutic Neuroradiology Unit, Clinical Department of Neurological and Motor Sciences, Azienda Ospedaliero-Universitaria Senese, 53100 Siena, Italy; 4 Unit of Diagnostic Imaging, Department of Emergency-Urgency, Azienda Ospedaliero-Universitaria Senese, 53100 Siena, Italy; 5 Institute of Radiology, Department of Diagnostics and Public Health, Policlinico GB Rossi, University of Verona, 37134 Verona, Italy; 6 Radiology Department, Fatebenefratelli Hospital, ASST Fatebenefratelli Sacco, 20121 Milan, Italy; 7 Unit of Diagnostic Imaging, Department of Radiological Sciences, Azienda Ospedaliero-Universitaria Senese, 53100 Siena, Italy; 8 Radiology Unit, Ospedale Santo Spirito, ASL AL Casale Monferrato, 15121 Alessandria, Italy; 9 Department of Clinical, Special and Dental Sciences, University Politecnica Delle Marche, 60126 Ancona, Italy; 10 Department of Biomedical Sciences and Morphological and Functional Imaging, University Hospital Messina, 98122 Messina, Italy; 11 Diagnostic Imaging Unit, Giovanni XXIII Hospital, Monastier di Treviso, Italy; 12 Radiology Service, Azienda Di Rilievo Nazionale Ed Alta Specializzazione (ARNAS) G.Brotzu, Cagliari, Italy; 13 Department of Biomedical Sciences, Morphological and Functional Images, University of Messina, Azienda Ospedaliera Universitaria Di Messina G.Martino, Messina, Italy; 14 Postgraduation School in Radiodiagnostics, Università Degli Studi Di Milano, Milan, Italy; 15 Department of Diagnostic Imaging, Radiology and Neuroradiology Unit, Fondazione Policlinico Universitario “A. Gemelli” IRCCS, Rome, Italy; 16 Unit of Diagnostic Imaging, Department of Medical, Surgical and Neuro Sciences and of Radiological Sciences, University of Siena, Azienda Ospedaliero-Universitaria Senese, Siena, Italy; 17 Department of Biomedical Sciences and Morphologic and Functional Imaging, University of Messina, Messina, Italy; 18 Neurological Institute-Foundation IRCCS “C. Mondino”, Pavia, Italy; 19 Department of Medicine and Surgery, Diagnostic Imaging, University of Perugia, Azienda Ospedaliera Universitaria Di Perugia, Perugia, Italy; 20 Diagnostic Imaging Unit, Humanitas Clinical Institute, Milan, Italy; 21 Department of Diagnostic and Interventional Radiology, Molinette Hospital, AOU Città Della Salute E Della Scienza, Radiologia 2, Turin, Italy; 22 Department of General Radiology, Azienda Ospedaliera Di Rilievo Nazionale E Di Alta Specialità San Giuseppe Moscati, Avellino, Italy; 23 Diagnostic Imaging Unit, ASL Benevento, Benevento, Italy; 24 Department of Diagnostic Imaging and Radioterapy, University of Perugia, Azienda Ospedaliera di Terni, Terni, Italy; 25 Diagnostic Imaging Unit, Candiolo Cancer Institute, Candiolo, Turin, Italy; 26 Section of Radiology - Department of Biomedicine, Neuroscience and Advanced Diagnostics (BiND), University Hospital Paolo Giaccone, Palermo, Italy; 27 Diagnostic Imaging Unit, Istituto Neurolesi Bonino-Pulejo IRCCS, Messina, Italy; 28 Department of Diagnostic Imaging, IRCCS Ospedale Policlinico San Martino, Genoa, Italy; 29 Department of Odontostomatologic and Specialized Clinical Sciences, Università Politecnica Delle Marche, Ancona, Italy; 30 Department of Diagnostic Imaging, Oncological Radiotherapy and Hematology, Fondazione Policlinico Universitario A. Gemelli IRCCS, Rome, Italy; 31 Department of Diagnostic Imaging, ASST Lodi, Lodi, Italy; 32 Department of Diagnostic Imaging, ASST-Spedali Civili of Brescia, Brescia, Italy; 33 Diagnostic Imaging Unit, Riccione Hospital, AUSL Romagna, Riccione, Italy; 34 Unit of Diagnostic Imaging, Department of Radiological Sciences, Azienda Ospedaliero-Universitaria Senese, , Siena, Italy; 35 Diagnostic Imaging Uniy, Conegliano Hospital, ULSS2, Conegliano, Treviso Italy; 36 Azienda Ospedaliera Di Rilevanza Nazionale Sant’Anna E San Sebastiano, Caserta, Italy; 37 Department of Radiological and Hematological Sciences, Section of Radiology, Universita’ Cattolica del Sacro Cuore, Rome, Italy; 38 Interdisciplinary Department of Medicine, Diagnostic Imaging, University of Bari, Azienda Ospedaliero-Universitaria Policlinico Di Bari, Bari, Italy; 39 Department of Radiology, Fondazione IRCCS Policlinico San Matteo, Pavia, Italy; 40 Diagnostic Imaging Unit, Department of Chirurgical Sciences, University of Torino, Azienda Ospedaliera Universitaria Città Della Salute, Turin, Italy; 41 Diagnostic Imaging Unit, San Camillo de’ Lellis Hospital, Rieti, Italy; 42 Emergency Radiology, Careggi University Hospital, Florence, Italy; 43 Diagnostic Imaging Department, Azienda Ospedaliero - Universitaria Policlinico Riuniti, Foggia, Italy; 44 Department of Translational Research, Academic Radiology, University of Pisa, Pisa, Italy; 45 Department of Radiology, Azienda Ospedaliera Dell’Alto Adige, Merano, Italy; 46 Diagnostic Imaging Unit, San Luigi Gonzaga Hospital, Orbassano, Turin, Italy; 47 Diagnostic and Interventional Radiology Unit, Ospedale Dei Castelli, ASL Roma6, Rome, Italy; 48 Padre Pio Bracciano Hospital, Asl Roma 4, Rome, Italy; 49 Department of Biomedical Sciences for Health, University of Milano, Milan, Italy; 50 Department of Radiology, IRCCS Policlinico San Donato, San Donato Milanese, Milan, Italy; 51 Department of Clinical Services, UOSD Radiologia, ASL Teramo, Teramo, Italy; 52 Radiology Unit, Department of Diagnostic Imaging, Michele E Pietro Ferrero Hospital, ASL Cuneo, Cuneo, Italy; 53 Department of Emergency Radiology, Cisanello Hospital, Pisa, Italy; 54 Department of Diagnostic and Interventional Radiology, ASST-Santi Paolo e Carlo, Milan, Italy; 55 Department of Radiology, Tatarella Hospital of Cerignola, ASL Foggia, Foggia, Italy; 56 Department of Emergency Radiology, Grand Hospital Antonio Cardarelli of Naples, Naples, Italy; 57 Diagnostic Imaging Unit, Azienda Ospedaliera Cosenza, Cosenza, Italy; 58 Radiology Department, University of Cagliari, Azienda Ospedaliero-Universitaria Di Cagliari, Monserrato, Cagliari Italy

**Keywords:** Computed Tomography, Dual-Energy computed tomography, National Recovery and Resilience Plan, Next Generation EU

## Abstract

**Objective:**

The main objective was to assess the geographical distribution and main areas of use of dual-energy CT (DECT) scanners in Italy prior to the implementation of the National Recovery and Resilience Plan (NRRP) within the Next Generation EU (NGEU) framework. Secondary objectives included the level of knowledge of DECT among radiologists and some possible solutions to maximize its use.

**Methods:**

Between February and March 2022, an anonymous questionnaire was conducted among the members of the Italian Society of Medical and Interventional Radiology (SIRM), using the Google form platform.

**Results:**

A total of 261 radiologists (mean age 41 ± 11.7 years) from 90 Italian cities participated in the questionnaire. 42% of them worked in an academic center, and diagnostic imaging was the main area of work for most participants (87%). Most radiologists had some knowledge gap, especially those who did not have a DECT scanner available in their hospital (48.3%). Weekly use of DECT protocols varied widely between centers. Oncological imaging (70.4%) was the main area of use. Radiologists use DECT to improve contrast resolution (65.9%), characterize materials (64.4%), and reduce the amount of contrast agent (60%). 91.1% of participants reported that DECT improved their diagnostic accuracy.

**Conclusion:**

DECT scanners have become increasingly available in Italy in recent years, and their number is expected to increase with the NRRP. There is a wide variation in their use and in the expertise of radiologists, leading to a huge untapped potential, that can be improved by specific training. A similar questionnaire will be conducted at the end of the NRRP in 2027.

**Supplementary Information:**

The online version contains supplementary material available at 10.1007/s11547-025-02044-5.

## Introduction

In 2020, the European Union (EU) took a bold step to help member states recover from the COVID-19 pandemic. As part of a comprehensive package of more than €2000 billion, the EU set aside €1200 billion for the long-term budget (2021–2027), known as the Multiannual Financial Framework, and around €800 billion for a temporary recovery effort, known as the Next Generation EU (NGEU), until 2026 [1, 2]. With the NGEU, Member States will have the opportunity to participate in the transformation of their countries by investing in six main policy areas including (1) green transition, (2) digital transformation, (3) social infrastructure and services, (4) advanced education and training in skills relevant to the future economy, (5) inclusive growth, research and development, and innovation for all, and finally (6) ensuring modern, efficient and accessible health services. In particular, the NGEU will enable European countries to (i) work all together to protect against health threats, (ii) invest more in research and innovation to develop vaccines and treatments, not only for infectious diseases but also for cancer, (iii) modernize health systems so that hospitals in every EU country have better access to new technologies and medical supplies, and (iv) fund training for Europe’s medical and health professionals [3, 4]. At the heart of the NGEU program are the Recovery and Resilience Facility (RRF), which provides loans that are made available to Member States, and the National Recovery and Resilience Plans (NRRP), through which each Member State accesses funding by drawing up plans following guidelines set by the European Commission. Dual-energy CT (DECT) is one of the most recent and promising technological innovations in computed tomography (CT), offering diagnostic advantages by reducing radiation and contrast agent doses [5–7]. DECT technologies include dual-source, single-source rapid switching, dual-layer, sequential (rotate-rotate), and single-source twin-beam [8]. Several radiological and neuroradiological applications of DECT have been described and well demonstrated, and the evidence of its benefits is accumulating to the point of implementation in guidelines [9–13]. Some other applications are still under investigation. However, even if the benefits for certain conditions are well established, DECT requires a skilled and determined radiologist, as the process of understanding, constructing, and interpreting the study can be laborious and time-consuming [14]. In Italy, this has contributed to generate both a lack of confidence in the radiologist's daily practice and a delay in implementation in hospitals. The first DECT scanners have been in operation in Italy since 2006, and the number of DECT scanners is expected to increase in many Italian hospitals. Therefore, in 2022, at the beginning of the Italian NRRP, the Subspecialty Section of Computed Tomography of the Italian Society of Medical and Interventional Radiology (SIRM) conducted a survey to collect the opinions of radiologists among the members of their society, to assess the actual availability of DECT scanners in Italy, to identify the available technologies, the level of knowledge of radiologists, and the patterns of daily use, and to identify any barriers to implementation and possible solutions to optimize use. A similar survey will be repeated in 2027, after the completion of the Italian NRRP, taking into account the scenario change that will be induced by the increased availability of photon-counting CT. The purpose of this paper is to present the results of the 2022 questionnaire.

## Methodology

The questionnaire consisted of 27 questions (general questions, n. 7, radiologists’ knowledge, n. 6, and DECT diffusion/use, n. 14) and was created using the free online platform Google Forms. A draft questionnaire was evaluated by three radiology residents and three radiologists, with 8, 9, and 20 years of experience, respectively, from our university centers, to improve the clarity and quality of the questions to achieve the objective. In addition, the reliability of the questionnaire was assessed by a pilot test among the board members of the Computed Tomography Subspecialty Section. A URL link to this anonymous questionnaire was emailed to SIRM members on February 01, 2022. A single reminder email with the URL link was sent to the same SIRM members on February 15, 2022. The questionnaire was also promoted through social networks such as Linkedin on the SIRM page and ran from February 01, 2022 to March 31, 2022.

**Fig. 1:**
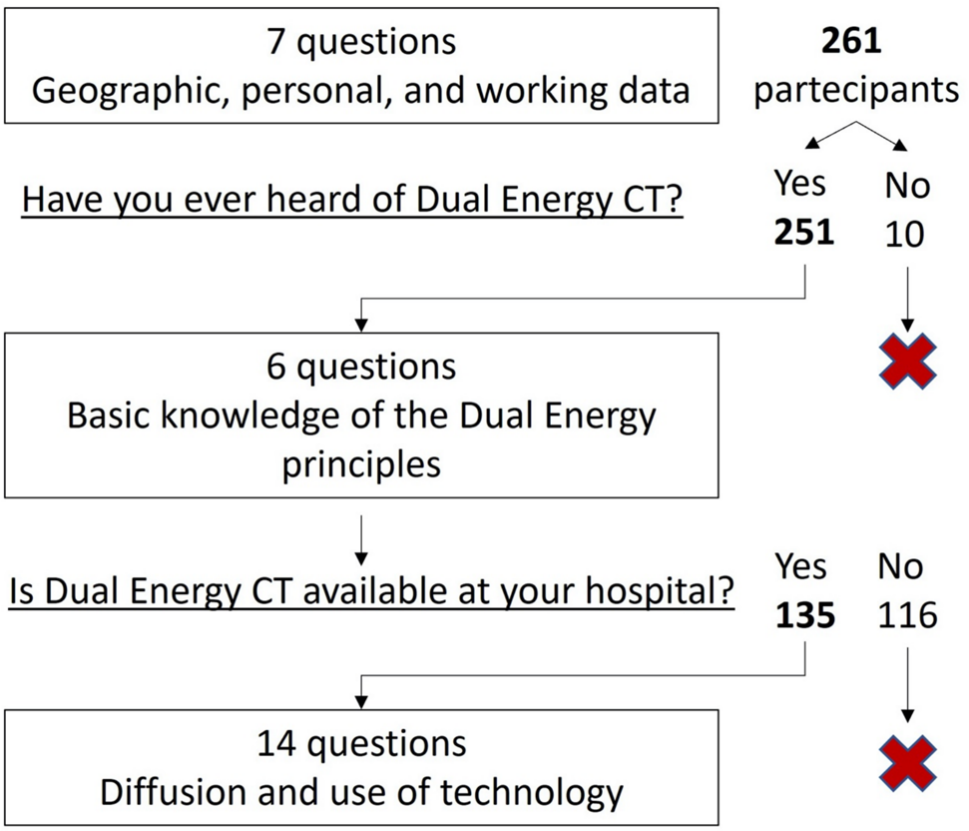
Flow of the questionnaire. Underlined questions were used to address the flow

**Fig. 2:**
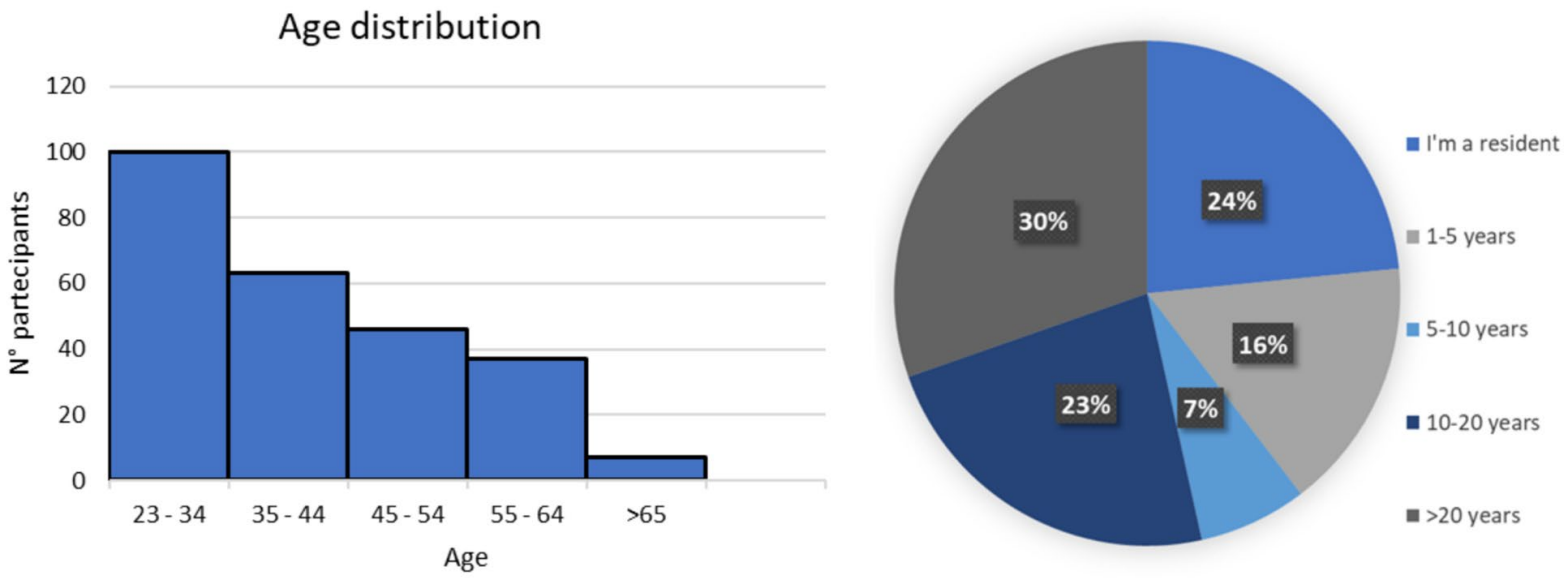
The mean age of the participants was 41 ± 11.7 years. Age distribution and years of experience of radiologists participating in the questionnaire

The questions were divided into three sections: (i) seven questions on geographical, personal, and main professional data of the participants; (ii) six questions on basic knowledge of DECT principles; (iii) 14 questions on diffusion and use of the technology (Fig. 1, Table S1). “Google My Maps” was used to create a map of the participants’ workplaces and the availability of DECT technology. Histograms, pie charts and bar charts were used to present the main findings. Exploratory statistical analyses were carried out to examine selected associations between the characteristics of the respondents and declared knowledge of or access to DECT. These results are included in the supplementary files. A total of 261 radiologists participated in the questionnaire, and responses were collected from 90 different cities in Italy. The age distribution and level of experience are shown in Fig 2. Among the participants, approximately 42% worked in an academic center, 18% in a non-academic hospital HUB, 17% in a non-academic hospital SPOKE, 10% in the private sector, approximately 8% in a Scientific Institute for Research, Hospitalization and Health Care (IRCCS), and 4% in other types of structures. Diagnostic imaging (87%) was the main area of work for the majority of participants, with 7% working in senology, 4% in neuroradiology, and 2% in interventional radiology.

### Geographical distribution and DECT availability in Italy

**Fig. 3:**
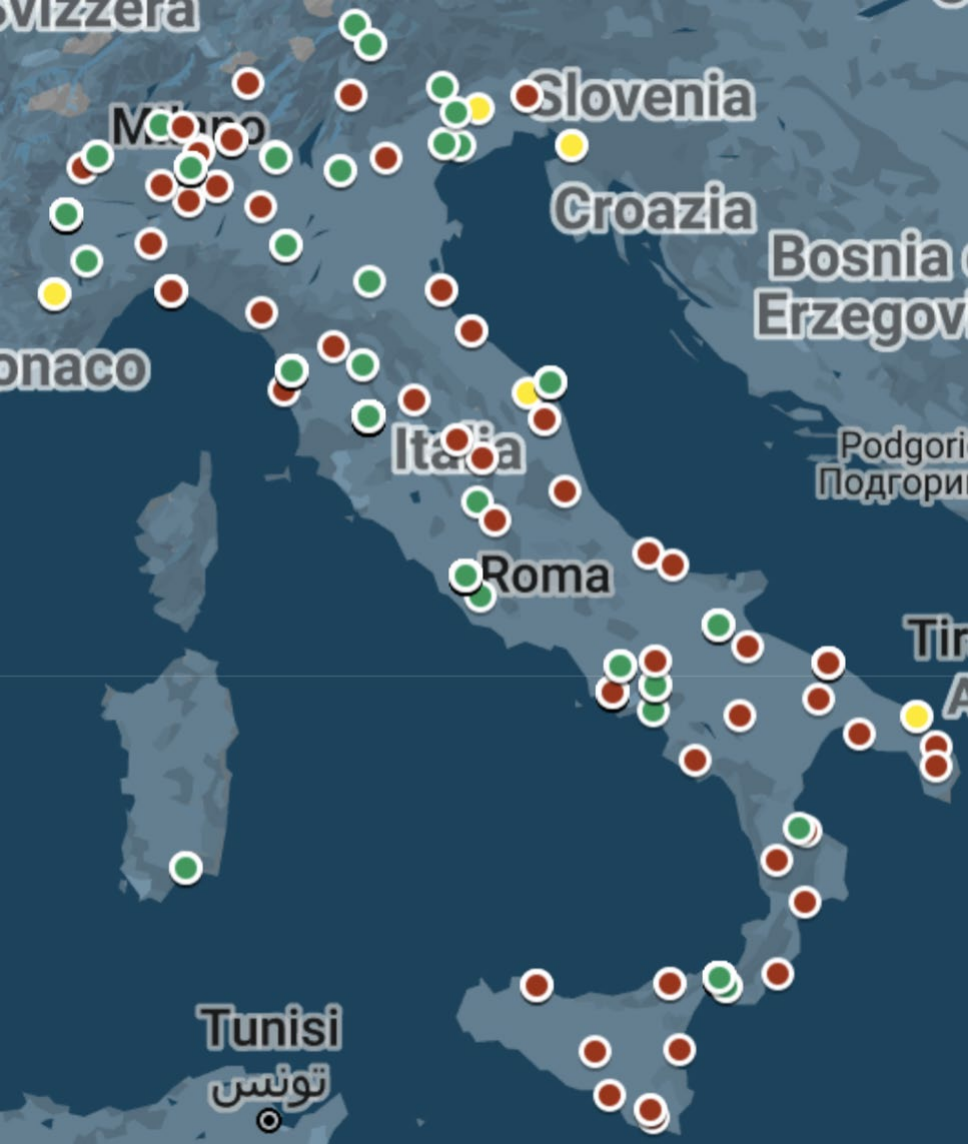
Green markers indicate hospitals where DECT is already available, and red markers indicate those where it is not. Yellow indicates hospitals where a DECT will be installed soon

**Fig. 4:**
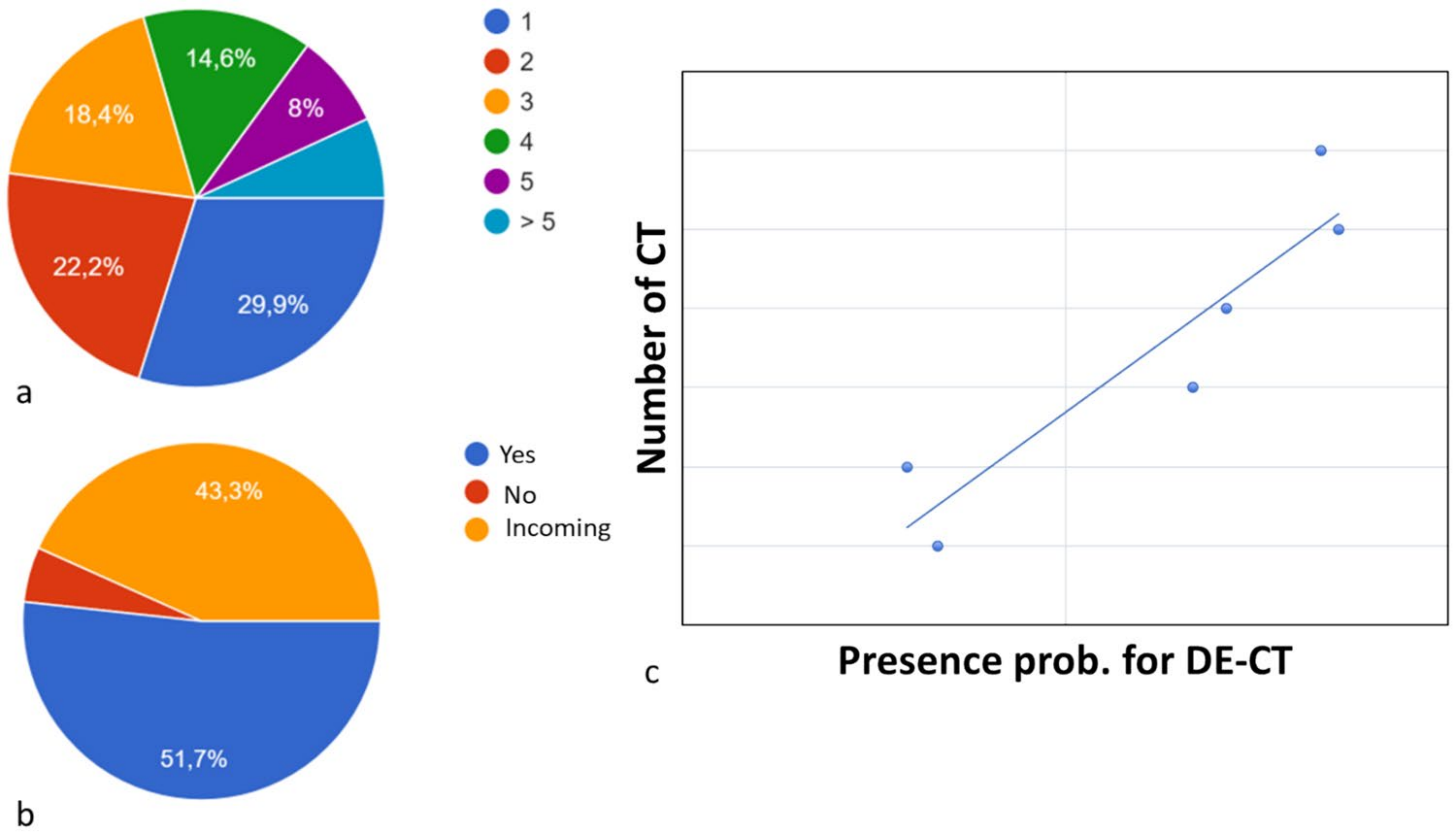
In **a** the number of CTs in the workplace; in **b** the availability of a DECT; in **c** note the linear relationship

**Fig. 5:**
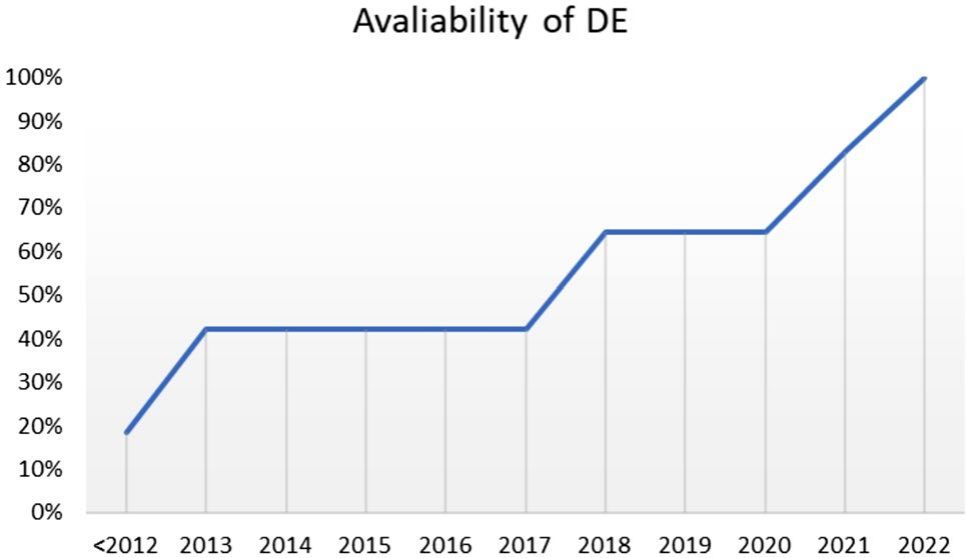
Evolution of the availability of DECT scanners over the last few years

DECT technology is available in 51.7% of the respondents’ workplaces and will be installed soon in 5% (Fig. 3). A significant difference was found when taking into account the availability of a DECT at the workplace (Odds Ratio = 2.8). There is a linear relationship between the total number of CTs installed and the availability of DECT technology. In most workplaces, the technology is only available for one scanner (66.7%), for two scanners in 22.2% of cases, and rarely for more than two scanners (Fig. 4). 58% of respondents said that DECT was installed less than 5 years before the date of the questionnaire, and 35% less than 2 years (Fig. 5). Ten respondents have more than one type of technology. Fifty-three (36.5%) respondents work in a center with a dual-source CT, 30 (20.7%) with single-source fast switching, 21 (14.5%) with a dual-layer, and 10 (7.6%) with other technologies. Thirty (20.7%) respondents did not know which technology was available at their institution.

### DECT radiologists' knowledge

Ten of the 261 participants (3.6%) had never heard of DECT. Table 1 shows the questions used to assess the knowledge of the remaining 251 participants. Only 16/251 (6%) of the participants answered all the proposed questions correctly, and the mean of the correct answers for each question was 34%. No significant differences were found in the mean of correct answers when taking into account different age groups, types of hospitals, and geographical origin. 256 out of 261 (98.1%) participants felt that specific DECT training could improve their knowledge and confidence in using DECT.

**Table 1:** Knowledge of radiologists

	Questions	Answers options	No. of answers
Q1	What is a possible definition of Dual Energy CT?	**DECT provides information about the composition of the material through which X-rays pass**	107 (43%)
		DECT uses two beams with different X-rays densities	80 (32%)
		DECT uses two different detectors next to each other	62 (25%)
		DECT can mainly improve spatial resolution	2 (1%)
Q2	What interaction effect with matter makes DECT possible?	**Photoelectric effect**	141 (56%)
		Compton effect	39 (16%)
		Photonuclear effect	14 (6%)
		I don't know	57 (23%)
Q3	What are the different technologies that make DECT possible?	**Dual-layer detector**	119 (47%)
		**Dual source**	229 (91%)
		**Sequential (rotate-rotate)**	56 (22%)
		**Single-source twin-beam**	76 (30%)
		**Single-source rapid switching**	112 (45%)
		Single-source dual booster	16 (6%)
		Single-source laser beam	2 (1%)
		Dual-IPA	8 (3%)
Q4	Which of these benefits can be achieved with DECT?	**Materials characterization**	181 (72%)
		**Improved visibility of hypervascularized lesions**	132 (53%)
		**Improvement of contrast resolution**	162 (65%)
		**Possibility to obtain a dose reduction**	195 (78%)
		**Possibility to reduce contrast volume**	177 (71%)
		Improvement of spatial resolution	45 (18%)
Q5	What are the benefits of Low-energy monoenergetic reconstructions?	**Improvement of contrast resolution**	159 (63%)
		**Reduction of contrast volume**	139 (55%)
		Improvement of spatial resolution	26 (10%)
		Metal artifact reduction	81 (32%)
Q6	What are the benefits of High-energy monoenergetic reconstructions?	Improvement of contrast resolution	73 (29%)
		Reduction of contrast volume	60 (24%)
		Improvement of spatial resolution	84 (33%)
		**Metal artifact reduction**	139 (55%)

Correct answers are shown in bold

### Current use of DECT

The weekly use of DECT protocols varies greatly from center to center. Notably, the number of DECT examinations performed per week varies from less than 5 (9.6% of participants) to more than 100 (5.2% of participants), while 34% of participants perform 20–100 DECT examinations per week. 28.9% of the participants are not able to estimate the number of DECT examinations performed per week. Table 2 shows the main areas in which DECT protocols are used, together with the percentages of participants. 91.1% (126/135) of the participants stated that DECT improved their diagnostic accuracy.

**Table 2:** Questions on the main areas of DECT use

	Questions	Answers options	% of answers
Q1	In which diagnostic area do you routinely use DECT?	Neuroradiology	18.5
		Musculoskeletal/rheumatic diseases	20.70
		Traumatic emergency	22.20
		Non-traumatic emergency	34.80
		CT angiography	45.20
		Oncologic diseases	70.40
Q2	In which non-traumatic emergency scenario do you use DECT?	I don’t perform urgency CT	17
		Acute abdomen	17
		Thoracic/abdominal hemorrhage	20
		Acute vascular pathology	25.90
		Intestinal ischemia	27.40
		Renal litiasis	53.30
		Pulmonary embolism	68.10
Q3	What musculoskeletal condition do you use DECT for?	I don’t do MSK imaging	36
		Extra-rachideal traumatism	8.90
		Metabolic diseases	8.90
		Infectious diseases	8.90
		Degenerative diseases	9.60
		Spinal traumatism	13.30
		Rheumatologic diseases	17.80
		Traumatic diseases	20
		Post-surgical (to reduce beam hardening)	39.30
		To analyze bone marrow edema	40.70
Q4	What clinical problems do you use DECT for?	To reduce beam hardening artifacts	60
		To reduce the amount of contrast media	60
		To characterize material/spectral analysis (lithiasis, edema, etc.)	64.40
		To improve contrast resolution	65.90
Q5	For which oncologic diseases do you use DECT?	I don’t do oncologic imaging	18.50
		Hematologic diseases	16.30
		Head–neck diseases	29.60
		Breast neoplasms	33.30
		Thoracic neoplasms	36.30
		Abdominal neoplasms	65.20
Q6	For which central nervous system diseases do you use DECT?	I don’t do CNS imaging	65
		Head injury: diagnosis	4.40
		Head injury: control	7.40
		Neuro-Oncology	14.10
		Ischemic ictus (control)	14.80
		Cerebral hemorrhage	15.60
		Ischemic ictus (diagnosis)	19.37

#### Emergency application

In the emergency setting, DECT is mainly used to diagnose and/or exclude pulmonary thromboembolism (68.1%) and, to a lesser extent, intestinal ischemia (27.4%) or active bleeding (20%). DECT has been shown to improve the assessment of arterial and venous vessels, even in a single scan and/or with a reduced contrast media dose or injection flow rate. As a result, the presence of thrombus and/or vessel occlusion can be detected with greater confidence, as can the presence of bleeding due to the increased contrast resolution in low-energy mono-energy reconstructions. Iodine map and low-energy monoenergetic reconstruction have been shown to improve the perception of perfusion differences in ischemic bowel loops [15]. It is known that DECT adds value to routine emergency department imaging by increasing diagnostic confidence, leading to a reduction in the number of recommended follow-up studies and a projected cost–benefit ratio, and it is, therefore, essential to improve its use according to the indications provided in the literature [15].

#### Oncological applications

According to the results of the questionnaire, in Italy, DECT is mainly used for oncological diseases (70.4%) and CT angiography (45.2%). Regarding oncological diseases, abdominal neoplasms (65.2%), thoracic neoplasm (36.3%), and breast cancer (33.3%) are the main indications for DECT; the result of the questionnaire showed a lower frequency in the evaluation of head and neck neoplasms (29.6%). Recently, several manuscripts have demonstrated the diagnostic utility of DECT for the detection and characterization of abdominal neoplasms [16]. The rationale is to increase the conspicuity of small hypervascular lesions, as in the case of hepatocellular carcinoma, intestinal or pancreatic neuroendocrine neoplasms, or to increase the contrast tissue to also facilitate the identification of hypovascular lesions. In addition, iodine quantification could be helpful in characterizing focal hypodense hepatic or renal lesions, overcoming the problem of pseudoenhancement CT artifacts [17, 18]. In thoracic applications [11], many studies have demonstrated the ability of DECT to characterize mediastinal masses, but also to evaluate and differentiate pathological mediastinal lymph nodes [19]; recently, a manuscript has shown that spectral CT analysis with iodine maps improves both quantitative and qualitative determination of pleural carcinomatosis [20]*.* The diagnostic performance of DECT in the locoregional staging of breast cancer is now well established in the literature [21] while a role for spectral analysis in the identification of preoperative sentinel lymph nodes has recently been proposed [22].

#### Neuroradiological applications

From the questionnaire results, DECT appears to be less popular in neuroradiology (main area of use: 18.5%), but many studies have addressed its unique role in defining some conditions, particularly in intracranial acute hemorrhagic lesions, follow-up of head trauma in patients undergoing intravenous iodinated contrast at diagnosis, and hemorrhagic transformation of acute ischemic stroke [23–28]. In the future, a specific survey on DECT in neuroradiology could be conducted to obtain more reliable data.

#### Musculoskeletal applications

The role of DECT in the analysis of bone marrow edema is one of the main reasons for using this technique in musculoskeletal disorders (40.7%), although DE can also be useful in reducing beam hardening artifacts in post-operative examinations (39.3%) [17, 29]. In recent years, DECT has been shown to allow calcium subtraction, facilitating visualization of bone marrow in the axial skeleton, and has the potential to improve the sensitivity for detecting diffuse bone marrow involvement compared with standard CT in patients with multiple myeloma [30, 31]. The visualization of bone marrow edema allows the problem of vertebral fractures to be solved by the reliably differentiating between recent and non-recent fractures [32]. It is also possible to diagnose stress fractures even in the absence of an intraspongiosa sclerotic reaction [33].

In rheumatology, especially in the evaluation of arthritic diseases such as gout, pseudogout, psoriatic arthritis, and rheumatoid arthritis, DECT plays a key role, especially in elderly and/or claustrophobic patients and/or with some contraindications to MRI [12].

## Discussion

DECT has many applications, primarily oncological, including the evaluation of abdominal neuroendocrine neoplasms, hepatocellular carcinoma, renal cell carcinoma, breast and thymic neoplasms, multiple myeloma, and pleural and peritoneal carcinosis [11, 16, 20–24, 34–39]. Non-oncological applications include assessment of bone edema in musculoskeletal disorders, and material characterization [11, 29, 32, 33, 40–42]. Neuroradiological applications [13] include differentiation between hemorrhage and iodinated contrast in acute hemorrhagic intracranial lesions [26, 27], head trauma [28, 43], and early follow-up of acute ischemic stroke treatment [44]. Studies in neuro-oncology are ongoing [45]. DECT also has several applications in pediatrics and emergency services, including hemorrhage, pulmonary embolism and intestinal ischemia [15, 46–51]. Other important clinical applications include the ability to overcome pseudoenhancement artifacts, due to iodine quantification, beam hardening artifacts from post-operative metallic implants, reduction of radiation exposure by avoiding non-contrast imaging, and a significant reduction in the amount of contrast agent required [7, 17]. This Italian questionnaire was prompted by the need to clarify and understand i) the availability of DECT scanners in Italy in 2022 (before the implementation of the NRRP), ii) the level of radiologists'knowledge of the principles and main indications of DECT, and finally iii) the possibility of identifying barriers to the use of this technique, and proposing possible solutions. To the best of our knowledge, this survey is the first to evaluate the patterns of use and the perceived benefits of DECT in the Italian territory. A total of 261 Italian radiologists, members of the SIRM, responded to this 27-question survey, and this number seems to demonstrate the huge increase in interest in DECT. Most notably, a previous 10-question survey, conducted in the UK [14] from July 20 to December 9, 2020, distributed to ten radiology departments affiliated to the Medical Physics Service (8 of which were known to have DECT scanners) and two national professional collaboration mailbases (Medical Physics and Engineering Mailbase with over 3200 subscribers, and CT Users Group including with over 200 members) received 25 responses [15 (60%) from radiographers and 10 (40%) from Medical Physics Experts (MPEs)/Clinical Scientists]. In 2017, the Society of Thoracic Radiology conducted a further survey in the USA: the focus was on the use of DECT in thoracic imaging, and a total of 104 responses were collected [52]. The Google Forms tool was used for its simplicity and ability to extract raw data for processing and analysis. The results describe a wide variation in the availability and use of DECT and even a lack of knowledge about DECT. However, only a tiny proportion of participants, i.e., 10/261 (3.6%), had never heard of DECT. Diagnostic imaging (87%) was the main area of work for most participants; only a small proportion of participants were neuroradiologists (4%) and interventional radiologists (2%), which may be related to the relatively small number of papers on DECT applications in these fields published in the literature in recent years.

DECT was available in more than half of the respondents’ institutions, more often in academic centers. As expected, the availability of DECT technology is related to the number of CT scanners in each hospital. The majority of DECT scanners had been installed less than 5 years before the date of the questionnaire, and a high proportion had been installed 1 or 2 years before, demonstrating the increasing diffusion of this technology in recent years.

The main clinical indications are well established in the literature, and DECT has become more widespread in Italy in recent years.

Weekly use of DECT varied widely between centers depending on speciality, but approximately 34% of radiologists reported performing more than 20 DECT examinations per week. This appears to be a higher level of DECT use than that reported in the 2020 UK survey [14].

Considering the general advantages of DECT and according to the questionnaire results, Italian radiologists use DECT to improve contrast resolution (65.9%), and thus lesion conspicuity, to characterize materials, with spectral analysis (64.4%), and to reduce contrast volume (60%) in nephropathic patients with mild renal failure.

Given the results of the knowledge section, it is encouraging that almost all participants have great confidence in the potential of DECT, with 91.1% of them (126/135) stating that DECT has improved their diagnostic accuracy. This seems to be in contrast to the 2020 UK survey [14], which at least addressed concerns about the usefulness of DECT (44%), lack of interest from radiologists (28%), and lack of requested DECT scans (11%). The survey conducted by the Society of Thoracic Radiology also found an extensive heterogeneity in the responses to the question on the perceived utility of DECT for the common indications in thoracic imaging: it was noted that only a small percentage of participating radiologists (between 22 and 41%) currently perform DECT, but express interest in doing so in the future [52].

However, as it is difficult to understand the basic concepts of DECT and therefore to use it to its full potential, almost all of our respondents (98.1%, 256/261) agreed that specific training on DECT could improve their knowledge. This should encourage radiologists to use this technology more safely and confidently. In addition, there are generally no pre-established protocols in the CT scanner, and DECT is usually activated at the request of the radiologist. The radiologist should therefore be skilled and determined, as the process of setting up the examination can be laborious and time-consuming. In addition, radiologists should sit next to the radiographer for each examination, and although this is good clinical practice, it may be difficult to achieve in some overburdened facilities. The possibility of having some common and optimized protocols recommended by radiological societies, including clinically useful reconstructions, could be a potential solution to increase and optimize use and to make radiologists more confident with this technique.

The technological scenario continues to evolve with the spectral imaging provided by photon-counting CT: photon-counting CT represents a major milestone in the development of a multi-energy CT system, offering significant improvements in tissue characterization, image quality, and spatial and spectral resolution. Although currently expensive and not widely available, it would be interesting to assess the knowledge and potential applications of this emerging technology [53, 54].

Limitations need to be considered. We know that this questionnaire still collected a relatively small number of participants and may only be partially representative of the wider population of Italian radiologists. Only a small number of interventional radiologists and neuroradiologists participated in the questionnaire, which limits speculation about these specific radiological populations.

## Conclusions

The availability of DECT scanners is increasing in the Italian territory, but their use varies widely and radiologists need specific training to improve their confidence. The main barriers to improving their use were identified as a lack of knowledge and a lack of standardized and shared protocols. Possible solutions to increase confidence include specific (i) training and retraining on DECT including its basics, benefits, and limitations, (ii) dedicated webinars, (iii) annual meetings, and (iv) the provision of shared and optimized clinical protocols by experts. In 2027, we will measure the evolution of DECT use in Italy.

## Supplementary Information

Below is the link to the electronic supplementary material.

Supplementary file1 (DOCX 30 kb)

## Data Availability

The datasets generated during the current study are available from the corresponding author on reasonable request.

## Abbreviations

CT: Computed Tomography
DECT: Dual-Energy computed tomography
SIRM: Italian Society of Medical and Interventional Radiology
IRCCS: Scientific Institute for Research, Hospitalization and Healthcare
NRRP: National Recovery and Resilience Plan
NGEU: Next Generation EU
EU: European Union
