# Supplementary material for: Italian radiologists and dual-energy CT: a state of the art from a shared document by the computed tomography subspecialty section of the Italian society of radiology
Source: Radiol Med. 2025 Jul 15;130(9):1396–408. doi: 10.1007/s11547-025-02044-5 (PMC12454489; doi:10.1007/s11547-025-02044-5)
Supplement: Supplementary file 1 — Supplementary file1 (DOCX 30 kb) [file 11547_2025_2044_MOESM1_ESM.docx]

**Supplementary files**

**Exploratory analyses**

Exploratory statistical analyses were performed to examine selected associations between respondent characteristics and declared DECT knowledge or access. Categorical associations were evaluated using chi-squared tests. Correlations between continuous variables were assessed using Spearman's rank correlation. A 6-point knowledge score was computed based on the number of correct responses to six DECT-related technical questions. Associations between this score and demographic/institutional variables were tested using Spearman's correlation and Mann–Whitney U tests.

Exploratory analyses showed that DECT knowledge scores (0–6 scale) were weakly but significantly correlated with both age (Spearman ρ = 0.25, p = 0.002) and years of experience (ρ = 0.23, p = 0.002), suggesting that increased professional seniority may be associated with greater familiarity. No significant differences in knowledge scores were observed across hospital types (p = 0.19), institutional DECT availability (p = 0.87), or declared interest in training (p = 0.09).

Additional comparisons revealed that familiarity with DECT did not vary significantly by geographic region (p = 1.000) or sub- specialization (p = 1.000), and DECT system availability was not associated with geographic area (p = 0.57). These findings suggest a broadly homogeneous distribution of DECT awareness and access across the sample.

These analyses are exploratory in nature and are presented solely to complement the descriptive purpose of the study.

| *PART 1: Personal information* | |
| --- | --- |
| How old are you? |  |
| How long have you been a radiology resident/specialist?  - I am a resident radiologist  - 1-5 years  - 5-10 years  - 10-20 years  - >20 years |  |
| What organization do you work for?  - Academic centre  - HUB non-academic hospital  - SPOKE non-academic hospital  - Private sector  - Scientific Institute for Research, Hospitalization and Healthcare (IRCCS)  - Other structure |  |
| In which city are you working? |  |
| What is your main area of occupation?  - Diagnostic imaging  - Diagnostic imaging in neuroradiology  - Interventional radiology  - Senology |  |
| Have you ever heard of dual-energy CT?  - Yes  - No |  |
| Could Dual Energy specific training improve your overall skills/education?  - Yes  - No |  |
| *PART 2: Knowledge (The correct answers are in bold)* |  |
| What is one possible definition for Dual Energy CT?  - **DECT gives information about the composition of the matter**  - DECT uses two beams with different X-rays density  - DECT uses two side-by-side different detector  - DECT can improve mainly spatial resolution |  |
| Which interaction with matter effect is mainly involved in DECT?  - **Photoelectric effect**  - Compton effect  - Photonuclear effect  - I don't know |  |
| What are the different technologies that make DECT possible?  - **Dual layer detector**  - **Dual source**  -  **Single source twin beam**  - **Single source Rapid Switching**  - Single source Dua Booster  - Single source laser beam  - Dual-ipa |  |
| Which of these are benefits of DECT?  - **Materials characterization**  - **Improvement of visibility for hypervascularized lesions**  -  **Improvement of contrast resolution**  - **Possibility to obtain a dose reduction**  - **Possibility to reduce contrast volume**  - Improvement of spatial resolution |  |
| What are the advantages of Low-energy monoenergetic reconstructions?  - **Improvement of contrast resolution**  - **Reduction of contrast volume**  - Improvement of spatial resolution  - Metal artefact reduction |  |
| What are the advantages of High-energy monoenergetic reconstructions?  - Improvement of contrast resolution  - Reduction of contrast volume  - Improvement of spatial resolution  - **Metal artifact reduction** |  |
| *PART 3: Diffusion of the DECT around Italy* |  |
| How many CT machines are there in the hospital/clinic where you work (count both DECT and conventional CT machines)?-  - 1  - 2  - 3  - 4  - 5  - >5 |  |
| Is DECT technology available in the hospital/clinic where you work?  - Yes  - No, work in progress on it  - No |  |
| *PART 4: Analysis of the distribution and current practice patterns of utilization* |  |
| How many CT scanners with DE technology are there in the hospital/clinic where you work?  - 1  - 2  - 3  - 4  - 5  - 6  - 7  - 8 |  |
| How long has your hospital had a DECT unit?  - 1 yr  - 1-2 yr  - 3-5 yr  - 5-10 yr  - >10 yr |  |
| Do you know how many DECT exams are performed each week?  - I don’t know  - <5 exams  - 5-10 exams  - 10-20 exams  - 20-50 exams  - 50-100 exams  - >100 exams |  |
| Which kind of DE technology is available?  - I don’t know  - Dual-layer (sandwich)  - Dual-source  - Sequential (rotate-rotate)  - Single-Source helical / twin-beam  - Single-source, rapid-switching |  |
| Where is DE technology installed?  - Diagnostic imaging department  - Emergency diagnostic imaging  - Neuroradiology department |  |
| In which clinical areas do you use DECT most?  - Traumatic emergency diagnostic imaging  - Not traumatic ER diagnostic imaging  - Neuroradiology  - Musculoskeletal/Rheumatology  - Oncology  - Vascular programmed study |  |
| What are the main clinical situations in which you use DECT in clinical practice?  - To reduce iodine contrast medium  - To reduce the beam-hardening artefact  - To obtain a better contrast resolution for vascular lesions  - To characterize matters with spectral analysis |  |
| In which of the following areas do you use DECT in your emergency imaging practice?  - acute abdomen  - chest-abdominal blood loss  - ischemic bowel  - kidney stones  - acute chest vascular pathology  - pulmonary embolism  - I don’t know |  |
| In which of the following areas do you use DECT in your oncologic imaging practice?  - Abdominal Neoplasms  - Breasts neoplasms  - Chest neoplasms  - Head-Neck district neoplasms  - Hematologic neoplasms  - I don’t know |  |
| In which of the following areas do you use DECT in your MSK imaging practice?  - Degenerative  - Bone edema  - Infections  - Metabolic disease  - Rheumatologic disease  - Traumas  - Post-traumatic (prosthesis and synthesis implants)  - Spinal trauma  - I don’t know |  |
| In which of the following areas do you use DECT in your neuroradiology practice?  - Cerebral hemorrhage  - Ischemic stroke (diagnosis)  - Ischemic stroke (follow-up)  - Neuro-oncology  - Skull brain trauma (diagnosis)  - Skull brain trauma (follow-up)  - I don’t know/I don’t usually practice neuroradiology |  |
| Do you think that the DECT has improved your diagnostic accuracy?  - Yes  - No |  |

**Table S1:** Full text of the questionnaire.
